# Roles of Key Ion Channels and Transport Proteins in Age-Related Hearing Loss

**DOI:** 10.3390/ijms22116158

**Published:** 2021-06-07

**Authors:** Parveen Bazard, Robert D. Frisina, Alejandro A. Acosta, Sneha Dasgupta, Mark A. Bauer, Xiaoxia Zhu, Bo Ding

**Affiliations:** 1 Department of Medical Engineering, College of Engineering, University of South Florida, Tampa, FL 33620, USA; parveen1@usf.edu (P.B.); aaacosta@usf.edu (A.A.A.); sdasgupta@usf.edu (S.D.); mabauer@usf.edu (M.A.B.); xiaoxiazhu@usf.edu (X.Z.); ding1@usf.edu (B.D.); 2 Global Center for Hearing and Speech Research, University of South Florida, Tampa, FL 33612, USA; 3 Department Communication Sciences and Disorders, College of Behavioral & Communication Sciences, Tampa, FL 33620, USA

**Keywords:** aging, cochlea, ion channels, presbycusis, age-related hearing loss, potassium channels, deafness, auditory, hearing, inner ear

## Abstract

The auditory system is a fascinating sensory organ that overall, converts sound signals to electrical signals of the nervous system. Initially, sound energy is converted to mechanical energy via amplification processes in the middle ear, followed by transduction of mechanical movements of the oval window into electrochemical signals in the cochlear hair cells, and finally, neural signals travel to the central auditory system, via the auditory division of the 8th cranial nerve. The majority of people above 60 years have some form of age-related hearing loss, also known as presbycusis. However, the biological mechanisms of presbycusis are complex and not yet fully delineated. In the present article, we highlight ion channels and transport proteins, which are integral for the proper functioning of the auditory system, facilitating the diffusion of various ions across auditory structures for signal transduction and processing. Like most other physiological systems, hearing abilities decline with age, hence, it is imperative to fully understand inner ear aging changes, so ion channel functions should be further investigated in the aging cochlea. In this review article, we discuss key various ion channels in the auditory system and how their functions change with age. Understanding the roles of ion channels in auditory processing could enhance the development of potential biotherapies for age-related hearing loss.

## 1. Introduction

Auditory system pathologies lead to hearing loss that can be categorized into two broad types: conductive or sensorineural hearing loss. Conductive hearing loss occurs due to deficits in the middle or outer ear, while sensorineural hearing loss occurs due to inner ear abnormalities. Age-related hearing loss (ARHL), also known as presbycusis, is the most common type of sensorineural hearing loss and is a highly prevalent communication disorder and neurodegenerative disease affecting the elderly worldwide. The majority of people above the age of 60 experience some degree of ARHL, impacting their auditory sensitivity and speech perception capabilities [1]; making it difficult for the elderly to communicate, and decreases the productivity and quality of their lives [2,3,4,5,6,7]. Presbycusis is a multifactorial process characterized by reduced sensitivity and speech understanding in noisy environments, slowed central processing of acoustic information, and impaired localization of sound sources, which can impart declines in music appreciation, and participation in family and social activities [8,9,10]. Deficits can occur at many points in the system, including inner ear structures: hair cells, auditory nerve, stria vascularis, marginal cells in the lateral wall, and others [8,11,12]. According to Schuknecht’s pioneering work (1969), there are four types of ARHL involving the cochlea: sensory, neural, stria/metabolic, and cochlear conductive [13,14]. Sensory presbycusis occurs due to degeneration of the organ of Corti, with major damage to outer hair cells. Neural presbycusis is a moderate-to-severe hearing loss at high frequencies, involving a significant decrease in speech recognition. It occurs due to loss of cochlear neurons. Strial or metabolic presbycusis is the loss of stria vascularis cells and functionality and shows hearing loss across all frequencies. Cochlear conductive hearing loss is characterized by presumed degenerations due to mechanical stiffness of the basal area of the cochlea. Later in 1993, Schuknecht and co-workers added two more types: mixed and indeterminate presbycusis. Mixed presbycusis is a combination of four types of hearing loss; and indeterminate occurs due to damage to the stereocilia tip links and their mechano-electrical channels [15]. They hypothesized that metabolic presbycusis, involving lateral wall and stria vascularis atrophy, is a predominant lesion in the inner ear, and the sensory cell and neural loss may be less prevalent [10,15,16]. Many animal models without noise history or genetic mutations support this conclusion [17,18,19]. Although recent evidence from human temporal bones calls this theory into question, and attributes more importance to the sensory and neural components of ARHL [20].

**Figure 1:**
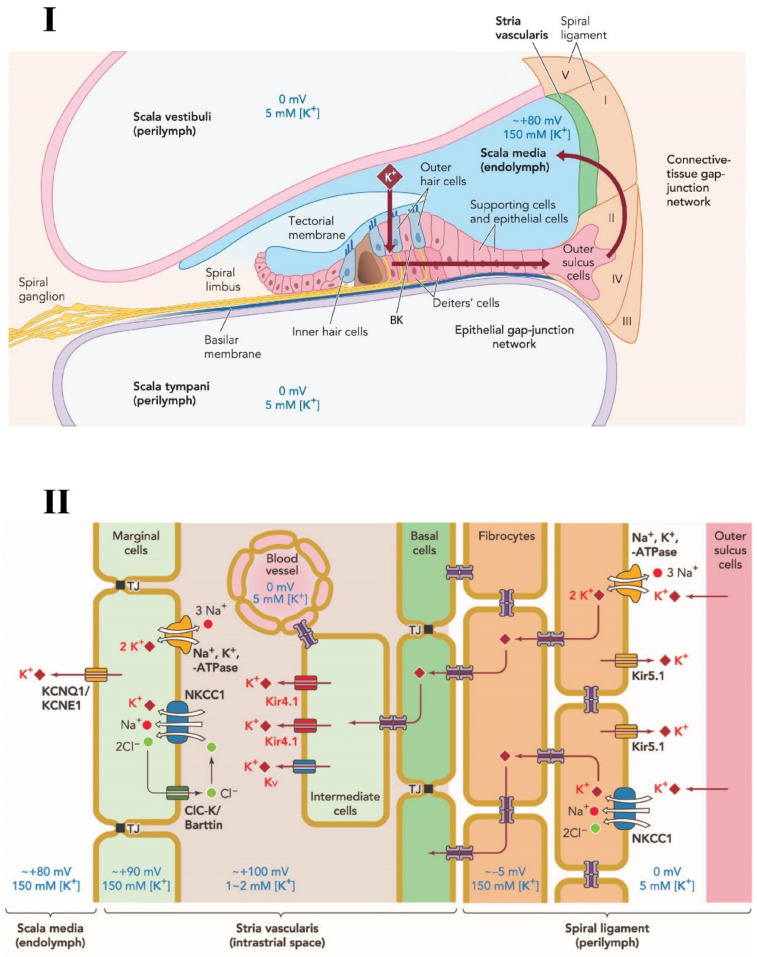
**Schematic diagram of potassium circulation, ion channels and transporters in the cochlear lateral wall**. (**I**) Potassium exits the hair cell and re-circulates into the endolymph of the scala media via various structures and ion channels in the supporting cells and lateral wall of the cochlea. (**II**) Various ion transporter channels; NKCC1; Na, K-ATPase; KCNQ1; Kir 4.1; Kir 5.1 and Kv, are expressed in the stria vascularis and spiral ligament that participate in potassium circulation and endocochlear potential generation. TJ: Tight Junctions. NKCC1, a key transporter in the cochlea, is expressed in spiral ligament and stria vascularis cells. Adapted from Hibino and Kurachi (2006) [25], with permission from the publisher.

The cochlea is a spiral structure that is made up of three fluid-filled compartments, scala media, scala tympani and scala vestibuli. Scala media is an unusual, high K^+^ and low Na^+^ concentration fluid zone that is sandwiched between two low K^+^ and high NaCl zones: scala tympani and scala vestibuli. Scala media’s unique extracellular solution, known as the endolymph, contains 150 mM K^+^ with a high, positive voltage (endocochlear potential: EP); ~ +80 to +100 mV (the highest positive voltage in the body). The apical surface and stereocilia of the sensory hair cells lie inside the endolymph. As sound stimuli occur, cation channels on the stereocilia open and endolymphatic K^+^ flows into the hair cells. This depolarization triggers an electrical excitation of the cells and leads to the release of neurotransmitter into the synaptic cleft of the hair cells and auditory neurons [21,22]. Dysfunction of the EP results in hearing loss or deafness since the highly positive EP is necessary for hair cell transduction. The EP is maintained by ionic fluxes in an intact ion channel transport system located on the lateral cochlear wall, which has two types of tissue: stria vascularis and spiral ligament [23,24]. Stria vascularis, an epithelial-like tissue, consisting of three types of cells: marginal, intermediate, and basal. These cells separate the endolymphatic space from the normal extracellular environment (high Na^+^, low K^+^) and facilitate selective passage of particular ions, fluids, and nutrients. Although previous studies have identified some proteins involved in the transport systems [23,25], more research is required for understanding the molecular elements and networks of the systems. In this review, we will discuss the scientific literature focusing on various ion channels and their roles in hearing loss, especially ARHL. The article also focuses on cochlear ion transporters—sodium-potassium-chloride transporters, sodium-potassium-ATPase and potassium channels. Calcium channels are not included here as there is not sufficient data available yet to summarize them meaningfully, in the context of age-related hearing loss. Figure 1 depicts the various channels expressed in the cochlea, especially in the cochlear lateral wall.

## 2. Sodium-Potassium-Chloride Cotransporter

Sodium-potassium-chloride, Na^+^-K^+^-2Cl^−^ (NKCC) cotransport protein is of particular importance in the regulation of osmotic homeostasis and ion concentration regulation in animal cells [26,27,28]. Specifically, this protein aids in electroneutral transport of Na^+^, K^+^ and Cl^−^ across the plasma membranes of cells [28,29,30]. In humans, the NKCC cotransporter has two isoforms: NKCC1 and NKCC2. These two isoforms are encoded by two different genes; namely, the *SLC12A2* gene, located on chromosome 5q23, and the *SLC12A1* gene, located on chromosome 15q15-q21, respectively. NKCC1 and NKCC2 monomers share around 60% sequence at protein levels, with NKCC2 slightly smaller than NKCC1 (NKCC1: 1212 amino acids, 131.4 kDa size; and NKCC2: 1099 amino acids, 121.3 kDa). NKCC1 has been experimentally observed to be a homodimer whose expression and composition remains relatively constant across different cell types [29,31,32,33,34]. The phosphorylation of the N terminus has been observed to produce movement in the C terminus as regulation of NKCC1 occurs. This suggest that the NKCC1 C terminus is involved in transport regulation which is amplified within dimeric pairs and could provide insight into the mechanisms of cation-coupled Cl^−^ cotransport as mediated by conformational changes [28,35].

While NKCC2 is localized only in the kidney, NKCC1 is primarily found in secretory epithelial cells where it is expressed in the basolateral membrane. However, northern probe studies have shown that NKCC1 can be found in the plasmalemma of many different cell types, including most non-epithelial cells [26,32,36]. NKCC1 is known to be physiologically important with key roles in cardiac, vascular, renal, hepatic, and sensory systems. It is also found within the nervous system, being present in the striatum, neocortex, hippocampus, dorsal root ganglia, and glia serving to regulate the generation, inhibition, and propagation of action potentials; as well as being expressed in the heart and skeletal muscle [26,37,38]. Within these systems, NKCC1’s main role is to regulate intracellular Cl- concentrations as well as overall cell volume [27,39,40,41]. To do this, NKCC1 facilitates the entry of Na^+^, K^+^ and Cl^−^ from interstitial fluid into cells. Specifically, when NKCC1 is activated, it allows the entry of Na^+^, K^+^, and 2Cl^−^ via cation-coupled chloride cotransport. This results in an electroneutral influx of ions into the cell [26,28,29,30,37,38]. For the regulation of cell volume, NKCC1 has an estimated turnover of 600 water molecules transported/molecule. This osmotic regulation is highly precise, as cells maintain their volume with an accuracy of ~2%. Furthermore, previous studies show that NKCC channel activation is linked with osmotic dysregulation in both shrunken and swollen cells through the activation of phosphatases and protein kinases [39,42,43,44,45].

Malfunction of NKCC1 can lead to complications, not least neurodegenerative diseases and attenuation of sensory system acuity like epilepsy, ataxia, hypertension, and hearing loss [46,47,48,49,50,51,52,53,54]. Also, studying interactions between enzyme activation and NKCC1 performance has led to the exploration of therapeutic countermeasures which show promising results. Regulation of NKCC1 function has been shown to improve the severity of Rett syndrome, Down’s syndrome, schizophrenia, Parkinson’s disease and other neurological conditions through GABAergic modulation [28].

### Presbycusis and NKCC1 (Sodium-Potassium-Chloride Cotransporter)

**Figure 2:**
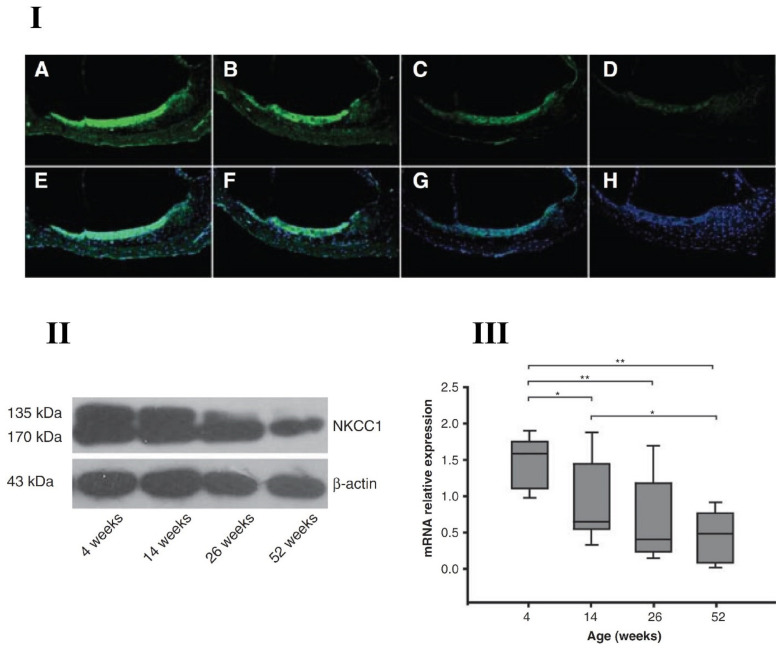
Aging decline of NKCC1 (sodium-potassium-chloride cotransporter) in C57BL/6J mouse cochlear lateral wall with four different age-group animals; 4 weeks old, 14 weeks old, 26 weeks old and 52 weeks old. (**I**) Age-related changes of NKCC1 protein stain with FITC (Fluorescein isothiocyanate, green fluorescence—top row) and the nuclei counter stained by DAPI (4′,6-diamidino-2-phenylindole, blue fluorescence—bottom row) for 4 animal groups at 400× magnification; A, E—4-week-old group; B,F—14-week-old group; C, G—26-week-old group and D, H—52-week-old group. (**II**,**III**) Western blotting and real-time polymerase chain reaction (RT-PCR) results from the four different age groups showed the gradual reduction in NKCC1 at both protein and gene expression levels. * *p* < 0.05, ** *p* < 0.01. From Liu et al. (2014) [55], with permission from the publisher.

Deterioration of the EP, linked to stria vascularis pathology, is a primary cause of ARHL. NKCC1 is expressed in marginal cells of the stria vascularis and plays a key role in maintaining the EP, by helping regulate K+ concentration in scala media. There are multiple reports demonstrating the role of NKCC1 in inner ear function and aging declines. For instance, Liu’s group [55] found that NKCC1 is mainly expressed in the cochlear lateral wall in C57BL/6J mice, and its expression levels decreased with age associated declines in hearing. Mice were divided into four different age groups: 4, 14, 26 and 52 weeks old. There was a steady decline in NKCC1 expression at both protein and gene levels with age; confirmed by immunofluorescence microscopy, Western blotting and quantitative real-time polymerase chain reaction (RT-PCR) techniques, as displayed in Figure 2. Frisina and colleagues reported similar ageing declines in NKCC1 expression for CBA/CaJ mice [47]. The mutation/disruption of NKCC1 led to deafness and structural damage in the inner ear of other mouse models [56,57]. Specifically, Delpire et al. [56] disrupted the *slc12a2* (NKCC1 gene) in mouse pups and found that the mutant mice were deaf. Significant damage was observed in the inner ear structures, such as collapse of Reissner’s membrane, shrinkage of stria vascularis, disorganization of the organ of Corti, including hair cell loss. Similarly, Flagella et al. [58] generated NKCC1 deficient mice and observed reduced growth, lower mean arterial blood pressure measured using a femoral artery catheter, and no auditory function, including collapse of inner ear structures. These studies demonstrated that NKCC1 is essential in transepithelial K+ movements for generation of potassium rich endolymph. In summary, NKCC1 plays a role in ARHL consistent with its participation in the production of the potassium-rich endolymph fluid, essential for inner ear function and sound transduction. For example, the application of the NKCC1 antagonist furosemide decreases the EP and, elevates the thresholds of auditory nerve fibers [59,60]. Through fitting the mean furosemide gerbil data to that of the human audiometric comparison, Schmiedt et al. [59] found the overall audiometric profiles of quite-aged and furosemide-treated gerbils and the human data are quite similar [59,61]. This finding strongly supports the hypothesis that ARHL in many humans is of metabolic origin. Given its critical involvement in aging changes in cochlear function, NKCC1 could be a potential therapeutic target for new ARHL treatments. Provocatively, the chronic treatment of aging CBA/CaJ mice with aldosterone, a natural occurring hormone that declines with age in mammals, prevents certain key aspects of ARHL (relative to aging control mice); i.e., aldosterone hormone therapy improved auditory brainstem response thresholds and improved survival of spiral ganglion neurons via inhibition of age-related downregulation of NKCC1 and apoptotic pathways [46,47,48,62].

## 3. Na^+^ K^+^-ATPase

Sodium-potassium adenosine triphosphate (Na^+^, K^+^-ATPase), also known as the Na^+^/K^+^ pump, is an enzyme primarily located in the cell membrane. It transports ions against a concentration gradient, i.e., sodium out of the cell and potassium into the cell. Na^+^, K^+^-ATPase is classified as a P-type ATPase. Furthermore, through evolutionary analysis and phylogenetic modeling, Na^+^, K^+^ -ATPase has been classified in the P2C family and its closet relative is H^+^, K^+^-ATPase [63]. These enzymes are P-type ATPase proteins composed of multiple subunits. Na^+^, K^+^ -ATPase is a heterodimeric protein that consists of two subunits that coexist in 1:1 stoichiometry (the catalytic subunits—α, and the regulatory subunits—β) and a FXYD protein [64,65].

The α-subunit is responsible for the catalytic character of the Na^+^/K^+^ pump by hydrolyzing ATP and transporting cations in and out of cells. This subunit is composed of approximately 1000 amino acid residues and has a mass of 110 kDa. Furthermore, four distinct isoforms of this subunit have been identified and their presence in varying tissues has been studied although their functional differences are not clear. The isoform α1 can be found in most tissues and is largely responsible for ionic transport against electrochemical gradients. Thus, in systems where large disparities in cation concentrations exist, α1 will be transported in either apical or basolateral locations to help maintain these gradients, e.g., renal and central nervous systems where Na^+^/K^+^ pumps maintain the unidirectional flow of ions. Within the renal system, Na^+^, K^+^-ATPase is present within the basolateral membrane of the tubular system in order to decrease loss of sodium. On the other hand, the Na^+^/K^+^ pump is localized at the apical region of the epithelial cells that line the choroid plexus. This allows them to maintain low sodium levels within the cerebrospinal fluid [66,67,68,69]. Due to their critical functions and sweeping presence, deletion or serious mutations of this isoform are mostly incompatible with life. However, in less extreme cases where somatic mutations of the gene occur, altered α1 function can lead to hormone imbalances as well as hypertension [68,70,71]. The α2 isoform, in contrast, is found predominantly within muscle, localized to the T-tubular membranes, and assists in the indirect regulation of Ca^2+^ due to its close proximity to the Na^+^/Ca^2+^ exchanger [72]. Within these muscles, α2 is proposed to help maintain long-lasting cardiac action potentials as well as adapt to dynamic muscle activity within somatic musculature [73,74,75]. Mutations in α2 can affect both transmembrane and cytoplasmic parts of the protein. The most studied mutations result in reduction or loss of function due to affected ion binding, ion transport, or compromised integrity of the pump. Meanwhile, other mutations impair the ability to transport the protein to the membrane [76,77]. The most prominent result of α2 mutations is the development of familial hemiplegic migraine (FHM) that is characterized by weakness in one side of the body during the attacks; and at-least 82 different mutations of the α2 gene are linked to FHM [78,79,80]. The next isoform, α3 is largely found within the nervous system; specifically, within dendrites and neuronal projections [81]. Due to low sodium affinity, the α3 pump is ideal for the restoration of sodium concentrations within dendritic spines that have a buildup of sodium as high as 100 mM due to summation of neural action potential inputs [68,82,83]. Mutations of this isoform have been consistently linked with the development of rapid-onset dystonia Parkinsonism (RDP), alternating hemiplegia of childhood (AHC), and CAPOS (cerebellar ataxia, areflexia, pes cavus, optic atrophy, and sensorineural hearing loss) [84,85,86]. These conditions were long considered to be different phenotypes of the same underlying genetic abnormality due to their similarity. However, considering further observations, they have recently been categorized independently. RDP is currently not treatable, as no therapeutic drug or therapy is available. This condition is triggered by a stressful event which results in an irreversible rostro-caudal gradient of dystonia and Parkinsonism which affect both the motor and psychiatric faculties of the patient [87]. Similarly, AHC is typically diagnosed early in childhood, and its attacks (lasting anywhere from minutes to days) are accompanied by full body unilateral weakness or paralysis, dystonia, nystagmus, and in extreme cases, epileptic seizures [88]. CAPOS attacks, lastly, are triggered by fevers and characterized by cerebellar ataxia, areflexia, pes cavus, optic atrophy, and sensorineural hearing loss, although nystagmus and hypotonia are also common symptoms [89]. Lastly, α4 isoforms are unique to spermatozoa [90]. These are also the most distinct of α isoforms when compared to other isoforms [91]. These isoforms, despite their significant inter-species differences, all share increased functionality on both hyper- and hypo-polarized sodium ion concentrations. This has been characterized as an evolutionary advantage as it makes the spermatozoa less susceptible to environmental insults [92].

The β-subunits act as molecular chaperones by aiding the integration and packing of the catalytic α subunit. These processes help the cell maintain proper function, facilitate routing to the plasma membrane, and protects against degradation [93]. These subunits, in humans, are expressed in three isoforms. They are composed of a small N-terminal containing only 30 amino acids, a TM helix, intracellular matrix, extracellular domain, and a C-terminal of roughly 240 amino acids [94]. All isoforms of β subunits are regarded as regulators of catalytic activity as well as playing key roles in cell motility, formation of tight junctions, mesenchymal cell transformation, and cancer [64]. The β1 isoform of this subunit stands out for its ability to react to high levels of oxidative stress by glutathionilation of a cysteine found in the middle of its transmembrane helix that is not present in any other isoform [95], which facilitates the Na^+^ K^+^ ATPase function. In parallel, β2 has the highest enzymatic effects by lowering the affinity for K+ and raising the affinity for extracellular Na^+^ in a more pronounced way than other isoforms. This discrepancy in kinetic properties is the result of a difference in tilt angles of the TM helix of the isoform. This isoform, furthermore, likely plays important roles in biological development, as β2 knock-out mice expire 17–18 days after birth. This has been theorized to occur as a result of improper function of important brain structures [96]. Lastly, the β3 isoform has been experimentally found to act analogously to β1 except for the additional glutathionilation. However, no confirmed links have yet been found between human β mutations and genetic diseases.

FXYD proteins are a family of seven proteins that are characterized by their FXYD sequence, two conserved glycine residues in the transmembrane domain and a serine residue. These proteins are thought to regulate and stabilize Na^+^, K^+^-ATPase through alteration of its affinity for Na^+^ [97,98]. These proteins have also been experimentally shown to have an effect on Na^+^, K^+^-ATPase potassium affinity [99]. Notably, FXYD1 (phospholemman) is overrepresented in the heart and has been shown to cover a range of functions which are integral for the presence of healthy cardiac activity. FXYD1 deficient mice demonstrate depressed contractile function and as well as increased cardiac mass; furthermore, this protein is associated with the modulation of the sodium/calcium exchanger as well as maximum voltage characterization of Na^+^, K^+^-ATPase [100,101,102,103,104]. FXYD2 and FXYD4 are also strongly associated with Na^+^, K^+^-ATPase and regulation of its sodium affinity. Specifically, FXYD2 decreases Na^+^, K^+^-ATPase’s affinity for Na^+^ while FXYD4 increases it [105]. Although these isoforms are not as vital for survival as FXYD1, FXYD4-deficient mice demonstrate impaired colonic Na^+^ transport, pointing to the physiological relevance of this isoform [106]. In contrast, FXYD2 is associated with resorption of Na^+^ within the nephron; yet, FXYD2-deficient mice did not show impaired renal function by virtue of compensatory mechanism within the kidneys [97]. Lastly, as is the case with β-subunits, no confirmed links have yet been found between human FXYD mutations and genetic diseases.

### Presbycusis and Na^+^, K^+^-ATPase

Na^+^, K^+^-ATPase is expressed in the lateral wall of the cochlea, specifically, in stria vascularis, and plays a crucial role in maintaining the EP. The direct evidence that Na, K-ATPase regulates the EP can be observed when ouabain (Na, K-ATPase inhibitor) is applied to the round window niche in guinea pigs, the EP declines monotonically, stabilizing at a minimum of +8.0 mV after approximately 30 min [107]. Degeneration of stria vascularis has been strongly linked to ARHL [108,109,110]. As an ion pump, Na^+^, K^+^-ATPase aids in the regulation and maintenance of the 80mV resting potential found in the scala media via recirculation of K^+^ from perilymph to endolymph [111]. This process charges the cochlear “battery” and preserves the voltage difference of scala media through the movement of K+ against a voltage and concentration gradient.

**Figure 3:**
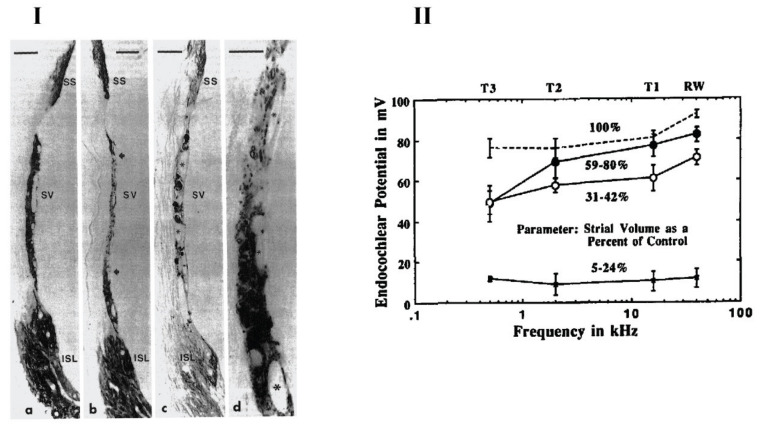
Na, K-ATPase expression in the cochlear lateral wall and endocochlear potential recordings for different age-group gerbils. (**I**) Middle turn sections of gerbil cochlea showing the Na, K-ATPase staining for a 6-month-old gerbil (a) and a 36-month-old gerbil (b–d). Various parts of lateral wall were stained for Na, K-ATPase expression; marginal cells in stria vascularis (SV), fibrocytes in the suprastrial zone (SS) and the inferior spiral ligament (ISL). There was a reduction in NA, K-ATPase immunostaining in the central portion of the SV (b—between arrows) as well as fibrocytes in the SS zone and ISL (c). Higher magnification of strial atrophy in part (b) is depicted in part (d). Strial capillaries (asterisks) were present in old age gerbil cochlea and remain patent (c and d). Scale Bars: a, b, c = 20 μM and d = 10 μM. Magnifications: a, b and c = ×800 and d = ×1800. (**II**) Mean values of endocochlear potentials of nine young animals (dotted line) and three groups of 35- to 38-months-old animals. The measurements were done at four positions—round window and three turns; T1, T2 and T3. Old animals were grouped based on the percentage of Na, K-ATPase immunostaining remaining as compared to the young adult control group. From Schulte and Schmiedt (1992) [118], with permission from the publisher.

Aging, however, causes a decline in the volume of the stria vascularis [110,112,113,114,115,116,117]. Consequently, the expression of Na^+^, K^+^-ATPase also becomes reduced within these structures. Schulte and Schmiedt [118] demonstrated the strong correlation between Na^+^, K^+^-ATPase expression and ARHL in gerbils. The study consisted of 41 Mongolian gerbils that were separated into four age groups: 5–12 months, 21–22 months, 29–31 months, and 35–38 months. The EP was recorded using a micropipette containing 0.5M KCl at four separate locations; the first being made at the scala media by passing the micropipette through the round window and basilar membrane. Subsequently, measurements were taken by drilling holes along the otic capsule’s overlying turns. The results showed that gerbils of more than 20 months of age had suffered significant loss of immunoreactive Na^+^, K^+^-ATPase in stria vascularis, located along both apical and basal turns of the cochlea. Furthermore, the regions of absent immunostaining grew larger with age strongly supporting the decay of both stria vascularis and Na^+^, K^+^-ATPase viability with ageing. The drop of EP magnitude was also reported in the same animals. Schulte and Schmiedt [118] concluded that around 64% of the decline in EP magnitude is linked to the loss of Na^+^, K^+^-ATPase in the stria vascularis. However, a significant level of redundancy was noted in the capacity for ion transport within the cochlea. It was observed that a 70% decrease in immunoreactive Na^+^, K^+^-ATPase in the stria vascularis induced a 25% decrease in the EP. However, for more than a 75% decrease, there was a rapid decline in EP magnitude. Figure 3 shows key findings of this study.

**Figure 4:**
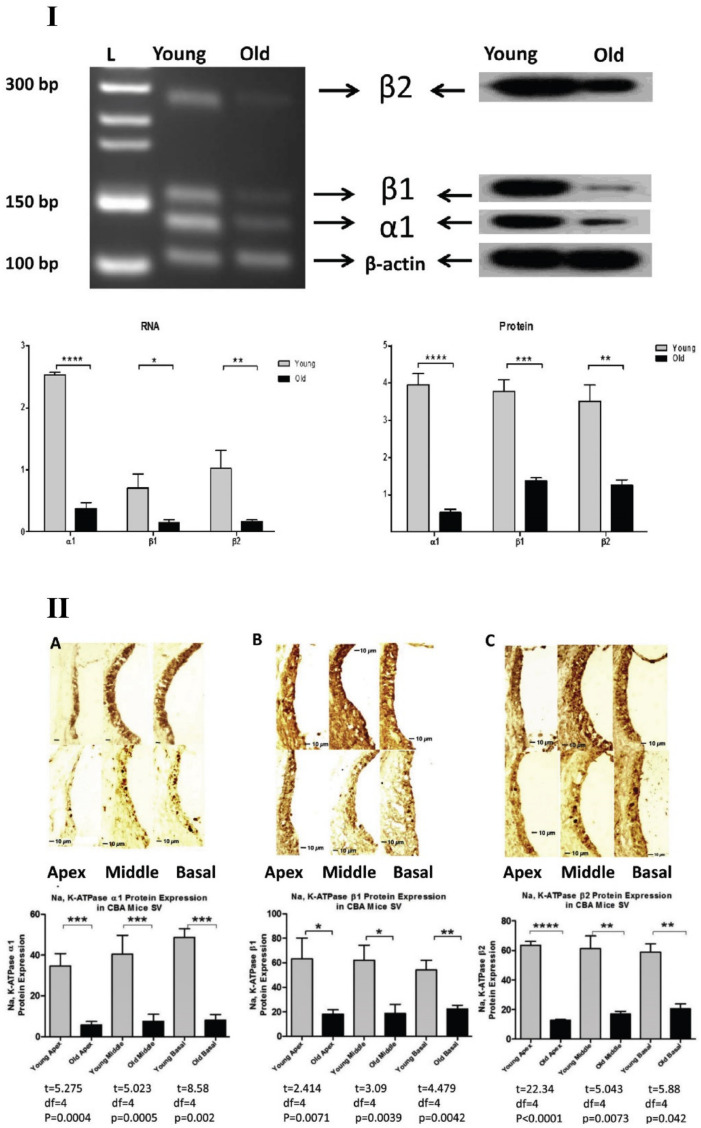
**Age-related decrease in Na, K-ATPase subunits in CBA/CaJ mouse cochlea**. (**I**) Protein lysate and mRNA extracts were analyzed for young adult (3 months) and old (30 months) CBA/CaJ mice using western blotting and RT-PCR techniques, respectively. There was significant decrease in α1, β1, β2 subunits of Na, K-ATPase at both protein and gene expression levels. (**II**) Cross sections of cochlea with immunostaining further confirmed the results using immunohistochemistry i.e., there was a significant decrease for Na, K-ATPase subunits α1, β1, β2 with aging in all three cochlear turns. * *p* < 0.05, ** *p* < 0.01, *** *p* < 0.005, **** *p* < 0.001. From Ding et al. (2018) [121], with permission from the publisher.

Hellier et al. [119] later corroborated relations between EP and ion channels/pumps by studying the optical density, quantity, and immunoreactive characteristics of Na^+^, K^+^-ATPase, as well as EP and stria vascularis density, in intermittently deafened guinea pigs. Additionally, Rarey and Lippincott’s study of both spontaneously hypertensive (SHR) and Wistar–Kyoto (WKY) rats further supports the relation between aging and Na^+^, K^+^-ATPase expression declines. Their study used 8 SHR and 9 WKY rats. The findings indicate that Na^+^, K^+^-ATPase immunostaining declined with age across both tested subunits (α1 and β1) for both animal models. The study further described the nature of relationships between the Na^+^/K^+^ pump and EP by correlating an increase in Na^+^, K^+^-ATPase staining with increased K^+^ concentrations within the endolymph [120]. Along similar lines, Ding et al. [121] studied the changes of Na^+^, K^+^-ATPase and its various subunits for aging in the CBA/CaJ mouse cochlea. Three isoforms of Na^+^, K^+^-ATPase; α1, β1 and β2 were detected, and there were aging declines in the expressions for all three isoforms at both protein and gene levels as shown in Figure 4. They also found that there was a preference for the α1-β1 heterodimer over α1-β2. α1-β1 interactions, observed in the young adult mouse cochlea were not found in old mice, suggesting that alterations in isoform interactions also play a critical role in functional declines of the aging cochlea.

In addition, the spiral ganglion cell density decreases 25% along the cochlear duct with aging and, interestingly, nerve fibers have large amounts of Na, K-ATPase [108]. In a ouabain mouse model, when ouabain was applied to the round window niche, all type I afferents underwent apoptosis, whereas the type II fibers that innervate the OHCs were preserved [122], similar to some age-related changes observed in the human cochlea. Clinically, the causative relations between strial degeneration and EP declines and loss of other cochlear cell types, such as hair cells and auditory nerve fibers, are not yet clear, since these changes cannot be studied directly in humans.

Although individual knockout of either NKCC1 or Na, K-ATPase leads to hearing loss, interestingly, Diaz et al. [123] reported that simultaneous deletion of NKCC1 and the α1 isoform of Na^+^, K^+^-ATPase in mice delayed the progression of ARHL. Also, the deletion of NKCC1 and α2–Na^+^, K^+^-ATPase helps protect hearing thresholds and the EP with aging. The mechanisms behind these counterintuitive findings need further investigation. For example, they could be due to crosstalk mechanisms between NKCC1 and Na-K-ATPase.

## 4. Potassium Channels

Potassium channels are ubiquitously present in most animal species except for some parasites [124]. There are approximately 70 distinct potassium channels in humans, many of which are present in cell membranes and are responsible for regulating the influx and efflux of K+ for excitable and non-excitable cells. Furthermore, these channels help determine the shape and timing of action potentials as well as the magnitude of the cell resting membrane potential. Potassium channels are categorized into the following classes depending on the structure and function of their transmembrane helixes: voltage gated (Kv), inward rectifying (Kir), tandem pore (K2P), and ligand gated (Kligand). Voltage-gated potassium channels are known to have six transmembrane helixes (TMs), inward rectifying channels contains two TMs, tandem-pore channels contain four TMs, and ligand-gated potassium channels are known to possess either two or six TMs [125,126].

Regardless of their categorization, all K^+^ channels are composed of a regulatory domain, responsible for sensing stimuli, and a pore-forming domain, responsible for ionic transport. The primary organization of K^+^ channels is a tetramer wherein each monomer has a pore-forming domain. Four pore-forming domains form a pore for ion flow [127]. Ion transport typically begins at the helical bundle from where ions enter the central water-filled cavity, then pass to the active site, which acts as a selectivity filter composed of four sequences, TVGYG in the 75–79th place of the genetic sequence, and then the ions end up in the extracellular entryway. During ion transport, the ions are hydrated at the central cavity, dehydrated at the selectivity filter, and finally rehydrated at the extracellular entryway [128]. Furthermore, due to the structure of the selectivity filter, four K^+^ ions can bind simultaneously to a tetramer allowing molecular interactions between potassium and oxygen; maximizing the rate at which this process occurs [127].

Potassium channels usually have three states: the activated state, inactivated state, and resting states. The channel’s state is dependent on electrochemical signaling and stimulation, and state changes occur through a process called gating. In the resting state, channels are closed and then open on stimulation, followed by inactivated states [129]. There are two kinds of gating mechanisms: the intracellular, which occurs at the spot where the inner helix bends; and the extracellular, which makes use of a selectivity filter [130]. These two mechanisms, or gates, are coupled; however, their coupling is dependent on the type of potassium channel. For instance, Kv channels are negatively linked, which favors the inactivated state. On the other hand, tandem-pore channels are positively linked, to favor the activated state [131].

Potassium channel abnormalities are associated with various diseases. For example, the selective filter (SF) of Kv channels play a key role in cardiac repolarization. Therefore, disturbance of the proper functionality of the SF in any of the states is associated with arrhythmias and sudden death [132]. Similarly, Kir channels, which are subdivided into seven subfamilies, are known to have a multitude of functionalities within human tissues. Kir channels can be categorized as homo- or hetero-tetramers and the specific Kir-6.x channels are known to aid in the control of insulin secretion within pancreatic β-cells [133]. Kir-6.x and their partners (SUR) have been linked to type 2 diabetes through dysregulation of insulin secretion. This link is so pronounced that drugs targeted to SUR are commonly used to treat type 2 diabetes [134]. Tandem-pore channels (K2P) are significantly abundant in both excitable and non-excitable cells. Therefore, it comes as no surprise that improper functionality of these channels has been related to a variety of complications ranging from cardiovascular, such as atrial fibrillation, to intestinal, such as Hirschsprung disease [135,136]. Also, Kligand, such as BKca, take part in many processes including generation of action potentials, tone modulation for blood vessels, hormone release, and neurotransmitter regulation [137,138]. In this review, we will focus on potassium channels related to hearing loss, specially, ARHL

### 4.1. KCNQ Channels

Like other potassium channels, KCNQ channels are composed of four subunits which constitute a pore, and each of these subunits consists of six transmembrane segments (S1–S6) where both the N and C terminus are located on the intracellular side of the membrane. Within these segments, the S4 contains the voltage sensor while the S5 and S6 form the pore domain along with a P-loop domain. Separately, the selectivity filter comprises four P-loops [139,140,141]. *KCNQ* is a gene family responsible for encoding five members of Kv7 channels: Kv 7.1–7.5 (KCNQ 1–5). Four of these channels (KCNQ 2–5) are expressed in neural systems and form the subunits of low-threshold Kv channels. These channels activate at low, sub-threshold voltages around −60 mV. KCNQ1, also coined KvLTQ1, is known to co-assemble to yield KCNE1 which is critical for the formation of cardiac delayed-rectifier-like potassium currents. Consequently, it has been found that mutations to KCNQ1 can lead to long-QT syndrome: a reduced capacity to repolarize the heart after each heartbeat [142,143,144,145]. Additionally, mutations to this channel have been associated with hearing loss, indicating that KCNQ1 plays a part in K^+^ recycling of the inner ear [140]. KCNQ2 and KCNQ3 share 40% homology with KCNQ1 and are thought to underlie the M current; a non-inactivating potassium current found in multiple neuronal cells [146,147,148]. These channels are commonly found within the nervous system and their mutations can lead to benign familial neonatal convulsions, a rare form of epilepsy [149,150]. The *KCNQ4* gene is theorized to encode the molecular correlate of I_K,n_ in outer hair cells and also, there is evidence that it is involved with I_K,L_ in Type 1 hair cells of the vestibular apparatus. Mutations of the KCNQ4 can lead to a form of deafness [140,141]. Furthermore, more recently identified KCNQ4, present in sensory neurons and the brainstem, can modulate stimulus-excitation coupling [151,152]. Lastly, KCNQ5 is most commonly expressed in brain and skeletal muscles and their mutations can cause retinopathy; however, it has also been observed in dorsal root ganglion neurons where they are hypothesized to aid regulation of pain sensitivity [141,153,154]. Furthermore, significant links have been found between KCNQ5 and KCNQ3, which has had interesting connotations as it suggests that KCNQ5 might also play a role in M current heterogeneity [155].

When compared to other Kv channels, KCNQ channels have an extended intracellular C-terminus which allows increased susceptibility of modulators and, therefore, greater variability of conformations and permeabilities. Neural KCNQs channels control somatic excitability, bursting, and neurotransmitter transport within the nervous system [141]. Within these roles, the binding capacity of KCNQ channels with modulators allows the channel to regulate ion flux through a variety of chemical agents. For example, KCNQ channels are known to alter potassium transport through modulation by Ca^2+^ [141], calmodulin [156,157], plasma membrane phosphoinositides [158,159], protein kinase C [160], SRC (sarcoma) tyrosine kinase and N-ethylmaleimide [161].

#### KCNQ and Presbycusis

KCNQ4 is related to proper hearing function, being expressed in both peripheral and central auditory systems. Its role in K+ recycling makes it a critical component of ion homeostasis and hair cell membrane potentials. As the auditory system is stimulated, K^+^ flows from the endolymph into the inner hair cells through mechanoreceptive ion channels in their stereocilia. These ions then start a recycling process through the basal membrane of the hair cells. It is during this step that KCNQ4 channels have a significant effect, as their locally increased staining density suggests that these channels are highly active within the basal portion of hair cells. As a result of this pivotal role, all missense and deletion mutations associated with KCNQ4 have been linked to a subtype of autosomal dominant non-syndromic sensorineural progressive hearing loss whose phenotype, like presbycusis, is characterized by reduced coding of higher frequencies [152,162,163,164,165]. Furthermore, Jung et al. [166] have shown a probable relation between a human *KCNQ4* mutation and late-onset hearing loss through electrophysiology assays of human embryonic kidney 293 cells. The cells were cultured in Dulbecco’s modified essential medium and transfected with wild-type (WT) or mutant *KCNQ4* plasmids as well as co-transfected with CHO-K1 cells for the purpose of electrophysiological assessment. They concluded that KCNQ4 variants around the pore-forming region of the channels significantly impaired K^+^ currents [166]. This, in turn, impairs K^+^ recycling, which is the primary patho-mechanism in progressive hearing loss [167,168]. However, the mutation of the *KCNQ4* gene is not synonymous with late onset or progressive hearing loss; instead, it acts as a marker and can be considered a risk factor that increases the probability of an individual developing such conditions. Van Eyken et al. [165] have also linked KCNQ4 channels directly to presbycusis through a study conducted on two separate elderly Caucasian populations. Statistical analysis was performed over 23 genotyped single nucleotide polymorphisms (SNPs) across *KCNQ4*. Within the first population, SNP9 and SNP15 yielded a significant *p*-value for disparity in auditory sensitivity of high frequencies (Z_high_) while SNP12 resulted in a significant *p*-value for a difference in the detection of low frequencies (Z_low_). On the other hand, the second population showed significant Z_high_ *p*-values for SNP18 and significant Z_low_ *p*-values for SNP6, SNP12, and SNP18. It is important to highlight that these SNPs are positively associated with presbycusis and furthermore, that they are all located within the same 13kb region within the *KCNQ4* gene. SNP9 and SNP18 were also related to high frequency presbycusis for females in both population groups [165].

Interestingly, *KCNQ1*, has also been linked to auditory function through the expression of its mRNA on the apical surface of stria vascularis marginal cells in the cochlear lateral wall. In addition, several channels expressed within the apical surface of vestibular dark cells, including a slowly activating, efflux inducing, Kv channel, are composed of KCNQ1 and KCNE1 [169]. Hence, impaired conductance of these channels has been linked to significant inner ear damage within *KCNE1*(−/−) mice [170] as well as its association with Jervell and Lange-Nielsen syndrome [141].

### 4.2. Inward Rectifying Channels

Inward rectifying potassium (K_ir_) channels exhibit different conductances for hyperpolarized or depolarized cell states. Distinctively, K_ir_ channels reveal higher conductance at hyperpolarization and lower conductance at depolarization for action potentials. This behavior, then, favors current flowing into the cell; hence, these channels are named as inward rectifying [171]. Furthermore, because altering the extracellular K^+^ levels results in shifting the peaks of the outward current, K_ir_ conductance is dependent on extracellular K^+^ concentration [171]. These ion channels are found in several types of tissue including cardiomyocytes, neurons, red blood cells, endothelial cells, glial cells, and epithelial cells [172,173,174,175,176]. K_ir_ channels are known to be comprised of 372–501 amino acids, wherein which there are two primary hydrophobic segments (M1 and M2) flanked by hydrophilic N and C termini. Additionally, although N termini are of relative constant length in all K_ir_ channels, C termini are known to fluctuate in length [177]. These channels are typically subdivided into seven subfamilies (K_ir_1.0–K_ir_7.0) when using molecular structure and electrophysiological properties as categorizing criteria [178]; or into four primary subgroups when considering biophysical characteristics. K_ir_ channels, hence, are categorized as follows: subgroup 1 (labeled Classical K_ir_ channels) contains subfamily K_ir_2.x; subgroup 2 (named G protein-gated K_ir_ channels) contains subfamily K_ir_3.x; subgroup 3 (ATP-sensitive K^+^ channels) contain subfamily K_ir_6.x; and lastly subgroup 4 (K^+^-transport channels) contain K_ir_1.x, K_ir_4.x, K_ir_5.x, and K_ir_7.x [179]. These channels are implicated in various diseases. Of these, K_ir_4.1 has been shown to be predominant within the inner ear of mammals where it is primarily expressed in the cochlear lateral wall, spiral ganglion cells, and supporting cells in the organ of Corti [180,181,182,183].

#### KCNJ10 and Presbycusis

Of the Kir Channels, Kir 4.1—encoded by the *KCNJ10* gene plays a critical role in development and maintenance of the cochlear EP, essential for functioning of the inner ear and sound transduction [179,182,183,184]. Kir 4.1 is expressed in various parts of the inner ear; such as the cochlear lateral wall, organ of Corti and spiral ganglion. Stria vascularis, the specialized area in the scala media lateral wall consists of several epithelial layers, including marginal, intermediate and basal cell regions. Marginal cells face the endolymph while basal cells are connected to the perilymph via the spiral ligament. The in-between isolated region is known as intrastrial space, having a low K+ concentration and a high positive potential (more than the EP by 10–15 mV). Kir 4.1 is located in the intermediate cells and facilitates the potassium diffusion across the apical membranes of intermediate cells. Kir 4.1 is essential to maintain the potassium concentration equilibrium across intermediate cells and the intrastrial space and generates the high transmembrane potential there [24,181,184,185,186,187]. Apart from lateral wall, Kir 4.1 channels are also found in supporting cells near outer hair cells and spiral ganglion neurons; consistent with its role in potassium recycling. Although much still needs to be learned about presbycusis and Kir 4.1 channels, *KCNJ10* reduced expression levels are related to various cochlear pathologies including ARHL. For instance, it has been demonstrated that *KCNJ10* knockout mice have profound deafness, including reduced acoustic startle responses, loss of EP, and significant degeneration in different inner ear structures. [188,189,190,191]. *KCNJ10* mutations are also associated with non-syndromic hearing loss [192].

**Figure 5:**
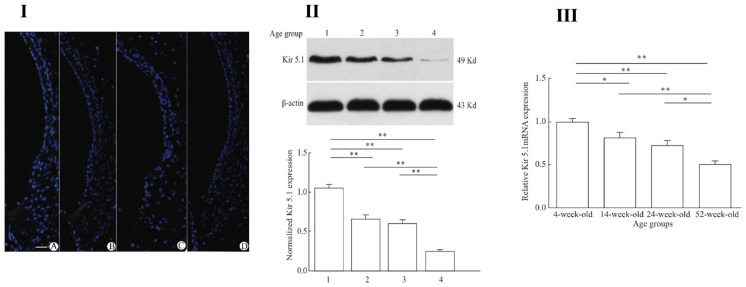
Age-related decrease of Kir 5.1 channels in the cochlear lateral wall of C57BL/6J mice. Cochlea from 4 different age-group C57BL/6J mice were analyzed using three different techniques; immunohistochemistry (**I**) scale bar = 10 µm, western blotting, (**II**), and RT-PCR (**III**). Four age groups tested: 4-week-old group, 14-week-old group, 24-week-old group, and 52-week-old group. There was a steady *decrease* in Kir 5.1 expressions levels at both protein and gene levels with age. * *p* < 0.05, ** *p* < 0.01. From Pan et al. (2016) [193], with permission from the publisher.

Pan et al. [193] demonstrated direct connections between inward rectifying channel 5.1 (Kir 5.1), encoded by gene *KCNJ16,* and presbycusis in ageing C57BL/6J mice. Forty mice were divided into four age groups: 4, 12, 24 and 32 weeks old. Kir 5.1 was observed in the cochlear lateral wall structures, including fibrocytes of spiral ligament. A decrease in expression of Kir 5.1 was observed with ageing at both protein and gene expression levels. Figure 5 presents this aging decline in Kir 5.1 channels. Overall, Kir channels, especially Kir 4.1 are vital for inner ear development and EP generation, and reduced expression of these can cause hearing loss. Further studies need to be conducted for detailed analysis of Kir channels in relation to presbycusis.

### 4.3. Ca^2+^-Activated K^+^ (BK) Channels

Large conductance Ca^2+^-activated K^+^ (BK) channels open in response to membrane depolarization and binding of intracellular Ca^2+^ and Mg^2^ [194,195,196,197]. Like the ligand and Kv channels, BK channels are composed of membrane-spanning domain and metal binding sites. Specifically, the membrane-spanning domain is comprised of a voltage sensor and a pore, while the metal binding sites form the cytosolic domain [198]. Like other K^+^ channels, BK channels are formed by four pore-forming subunits which are encoded by a singular *Slo1* gene. These channels then achieve functional diversity through splicing of the *Slo1* mRNA, or modulation by β subunits (5–10). This *Slo1* gene contains three primary structural domains labeled voltage-sensing domain (VSD), pore-gate domain (PGD), and cytosolic domain. Each of these domains has a specific and distinct function. VSD senses the membrane potential and the PGS controls permeation of K+ through conformational changes, and the cytosolic domain is sensitive to Ca^2+^ [198]. The VSD and PGD (or membrane spanning domain) are composed of transmembrane segments S1–S4 and S5–S6, respectively [199]. Unlike many other K^+^ channels, BK channels only contain one charged residue (Arg213) that contributes to voltage sensing within the S4 helix [200]. Furthermore, BK channels contain an additional S0 segment required for β subunit modulation; which can modify voltage sensitivity [201,202,203]. Alternatively, the cytosolic domain is made up of two RCK (regulator of K^+^ conductance) domains named RCK1 and RCK2 [204]. Furthermore, the cytosolic domain contains two Ca^2+^ binding sites which are considered to express a high level of affinity. The first of these sites, is located at position Asp362/Asp367 lies within RCK1 while the second Ca^2+^ biding site is located in a region labeled the Ca^2+^ bowl within RCK2 [204,205,206,207,208]. BK channels also have an Mg^2+^ binding site situated at the interface of the VSD and the cytosolic domain [209].

Currently accepted models for activation of these channels propose a conformational change induced by a ligand binding. This new conformation “pulls” the activation gate open with a mechanism called the “tugging model” [210,211,212,213]. However, in BK channels Mg^2+^ activates the channel differently. In Mg^2+^ interactions, the channel is activated by pushing the voltage sensor through an electrostatic interaction between side chains in different structural domains. This mechanism is coined a “nudging model” [198]. Interestingly, Ca^2+^ binding has shown to occur through more complex mechanisms, and it has been speculated that both of these high-affinity binding sites may interact through distinct mechanisms [198].

BK channel dysfunction has also been linked to several neurological disorders such as schizophrenia, and antipsychotic drugs improving K^+^ conductance contribute to therapeutics [214]. For example, schizophrenic patients were found to have significantly lower levels of mRNA expression for BK channels. Likewise, Laumonnier et al. were able to link mental retardation and autism to haploinsufficiency of the *Slo1* gene, which encodes for BK channels, as well as low levels of BK channel expression [215]. Furthermore, some mutations of the *Slo1* gene, occurring in the β3 and β4 subunits have been shown to increase Ca^2+^ sensitivity of BK channels. This increased sensitivity has been theorized to reduce action potential thresholds as well as reduce latency of nerves; thus, increasing the firing rate of neurons [216]. Brenner and coworkers study supports this, as β4 knockout mice exhibit neuronal hyper-excitability and epilepsy [217].

#### BK Channels and Presbycusis

BK channels expressed in cochlear hair cells contribute significantly to these cell’s outward K^+^ conductance [218]. The fast rate of activation of BK channels set them apart from other slower-acting ion channels [219]. Expression of BK channels within the mammalian inner ears suggests not only that BK channels contribute to high-frequency hearing in mammals, but also that these channels might be a necessary specialization for the expanded auditory frequency range of mammals [218]. The rapidly activating BK currents allow for lower input resistances as well as shorter membrane time constants which provide better voltage control during rapid changes that are more prevalent at higher sound frequencies. Rohman et al. [220], for example, showed that BK channels, compared to SK channels, provide a larger and faster conductance change in response to acetylcholine, increasing the gain and speed of efferent inhibition [220]. Moreover, an increase of BK channel expression coupled with a downregulation of voltage-gated calcium channels has been proposed to convert immature inner hair cells, showing calcium-driven responses, into mature inner hair cells, displaying graded receptor potential changes in response to K+ influx [221]. Therefore, the expression of BK channels appears to be a defining biophysical indicator of the mature inner hair cell.

Given the roles of BK channels for inner hair cells, hypotheses have been proposed for the auditory capabilities of *Slo1*^−/−^ mice. Specifically, these mice appear to be more susceptible to noise-induced hearing loss as well as presbycusis [222,223]. Consistent with this, Ruttiger and co-workers found that mice with deleted BKα-subunits showed progressive hearing loss. This study observed the effects of BKα-subunit deletion on knockout mouse frequency thresholds compared to wild-type mice. There was no effect of BK—β1 subunit deletion on hearing function. For the first 4 weeks of life, cochlear function was equal in both groups. However, by week 8 a statistically significant difference was observable in these two groups where the BKα^-^ mice had poorer hearing than their wild-type counterparts. This difference continued to grow for the duration of the study reaching its maximum around 12–17 weeks [223]. It is also important to point out that the disparity in thresholds was most pronounced at higher frequencies which, when coupled with the late onset, strongly correlates with the presentation of presbycusis. Similar results were reported by Kurt et al. [224] demonstrating the critical role of BK channels in auditory processing. Along similar lines, the deletions and mutations of *BKβ2* and *BKβ4* have been linked to hearing loss through BK channel inactivation [225,226,227,228]. Given the critical role BK plays in hearing function, it is possible that it plays a role in ARHL as well [229]. However, there is still little evidence to support correlation between BK channels and presbycusis. Further studies are needed to test this hypothesis.

### 4.4. Other Potassium Channels Related to Presbycusis

Transient potassium current channels (IA channels) are a family with a basic function- they are opened by depolarization following hyperpolarization. By increasing the interval between action potentials, they help a neuron to fire repetitively and accurately at low frequencies. These channels are localized in many parts of the central nervous system (CNS) and a main role is to modulate feedforward and feedback inhibition along the dendroaxonic axis [230]. However, despite prominence in the CNS, the IA channels’ role in the processing of the aged cochlea remains understudied. Avenues for further research that address specific gaps in our knowledge about this channel family in the aging auditory system are needed. Four major classes of K^+^ channels exist in the central auditory system: calcium-activated, inwardly rectifying, leak, and Kv. These channels usually share a common homotetrameric structure with all α-subunits being identical, but a few of them are heterotetrameric with two or more non-identical α-subunits [231,232]. The α-subunits are named as Kv1.4, Kv3.3, Kv3.4, Kv4.1, Kv4.2, and Kv4.3, which are classified as discrete families based on sequence similarity form and ion pore and infrastructure of the channel. The α-subunits have the fast-kinetic properties of IA channels [233,234]. In addition, the β-subunits and other auxiliary subunits are subunits for modulating the biophysical properties and functions of IA channels [233,235,236,237].

Among various Kv channels, Kv1.1 and Kv3.1 are of particular interest for auditory function as both are expressed highly in the brainstem auditory system. Within the central auditory system, the medial nucleus of the trapezoidal body (MNTB) plays important roles in temporal processing and tonotopic coding of auditory signals in the brainstem [238,239,240,241]. The MNTB within the superior olivary complex (SOC) is associated with gradients of Kv1.1 and Kv3.1 expression. For example, Kv3.1 exhibits its highest expression at the medial end corresponding to high-frequency sound processing where high-frequency spike firing is required, whereas it displays lower expression in the lateral end which is associated with lower-frequency sound coding. Similar gradients of Kv1.1 also exist along the tonotopic axis of MNTB, though density localization (medial vs. lateral expression levels) vary per rodent model used [239,242,243]. It is known that Kv3.1 channel transcriptional modification is controlled by Ca^2+^-cAMP response element-binding protein (CREB) transcription factor, i.e., action potential stimulation is needed to regulate the expression of these proteins where these Kv channels allow calcium influx due to depolarization events [244].

In temporal processing, Kv3.1 contributes to the rapidly activating high-voltage activation currents, assisting in cell repolarization and action potential timing reduction, thus enabling the high frequency spike coding. Conversely, Kv1.1 contributes to rapidly activating low-voltage activation currents by reducing the cell membrane time constant and minimizing the temporal summation of inputs, thus, enabling the ongoing time coding of sound signals [245,246,247]. Both channels, Kv3.1 and Kv1.1 are also expressed in other parts of the central auditory system that code the tonotopic signal of the cochlea along the auditory pathway e.g., cochlear nuclei and inferior colliculi [239,241,243,248,249,250].

Declines in temporal processing are a hallmark of ARHL clinically and in animal models, and Kv3.1 and Kv1.1 appear to play a role in ARHL due to changing patterns of expression/function with age. For instance, Frisina and coworkers reported declines in Kv3.1b, a subunit of Kv3.1 channels, in various parts of the CBA/CaJ mouse auditory system. In this study, four different age groups were used: 3–4 months, 15 months, 24–26 months and 29–34 months. Significant reductions in expression levels were observed by the age of 15 months in the MNTB, anteroventral cochlear nucleus (AVCN) and lateral superior olive (35%, 26% and 23%, respectively), notably limited to the neuropil of the axons. Cell density decline was also observed in other parts, the medial olivocochlear (MOC) feedback system such as the superior paraolivary nucleus, ventral nucleus of the trapezoid body and lateral nucleus of trapezoid body (24%, 29% and 26%, respectively) with no age-related changes observed in other parts of cochlear nucleus or inferior colliculus [251].

Interestingly, these age-related declines in the MOC were also observed in physiological measurements—contralateral suppressions of distortion product otoacoustic emissions (DPOAEs), where declines were observed in CBA/CaJ subject groups by 15 months of age. These findings were further confirmed in young (6–11 weeks old) homozygous Kv3.1b knockout mice where almost no contralateral suppression of DPOAEs was observed, in contrast to heterozygous and wild type groups with robust contralateral suppression. However, no effect was seen in auditory brainstem responses (ABRs) and DPOAE amplitudes. Taken together, these results indicate a strong correlation between Kv 3.1b age-linked reductions in expression levels and the MOC feedback system declines, while indicating that Kv3.1b is not vital for the overall sensitivity of outer hair cell functions [251]. Further corroborating the importance of Kv3.1 in ARHL, Chambers et al. [252] modulated this channel using the pharmacology compound AUT00063, and reported a reduction in action potential timing variability and improved temporal coding in mice.

Similar histological studies by Jung et al. [253] found that among all auditory brainstem regions, only PVCN revealed age-related changes in 24- to 29-month-old Sprague–Dawley rats as compared to 4- to 6-month-old young adult animals. Immunohistochemistry analysis revealed increased Kv1.1 immunoreactivity in the octopus cell bodies of this region with aging, whereas the expression intensity decreased in the neuropil. In contrast, decreased intensity of Kv3.1 was observed in the octopus cells and neuropil of aged PVCN. This suggests that these changes may affect ion channel activity and signal processing in the central auditory system with progressive age. Likewise, expression changes of Kv3.1 in aging C57BL/6 mouse models can also be seen in the MNTB, where loss of Kv3.1 tonotopicity and alterations in cAMP response element-binding protein signaling was observed [244]. An apparent Kv3.1 gradient was found in 6-week-old young mice with no gradient observed for CREB. However, older, hearing-impaired 8-month-old mice showed an abolished Kv3.1 gradient with a simultaneous decline in overall CREB expression and change in distribution patterns of the activated, phosphorylated form of CREB along the tonotopic axis of MNTB. These findings support the hypothesis that ongoing activity to the auditory brainstem neurons is needed to maintain Kv3.1 tonotopicity through the CREB pathway with age [244]. Further confirmation of this required brainstem activity can be seen in a study by the Leao group [242] where congenitally deaf pups were analyzed for Kv1.1 and Kv3.1 expression. It was seen that these congenitally deaf pups did not show Kv3.1 and Kv1.1 gradients as compared to normal hearing pups, most likely due to the absence of spontaneous auditory nerve activity. These findings demonstrate the role of Kv channels in developmental stages as well as potential therapeutic targets for temporal processing deficits, including aging deficits.

## 5. Summary and Conclusions

The ion channels and transport proteins associated with cochlear K^+^ processing and recycling pathways are essential for normal hearing. For example, age-related changes in these channels usually lead to hearing disorders, including temporal processing deficits. Ion channels involved in K^+^ flux—including the elusive transduction channels of hair cells, and ion channels in neurons and stria vascularis—are included in this group. To better understand the biological mechanisms and interactions among these K+ channels and co-transporters, like NKCC1, Na^+^, K^+^ ATPase, is an enthralling research challenge. A next important step is to understand in more detail the normal function of each channel expressed in the auditory system, and importantly, their interactions with each other with aging. Virus-guided infection of cochlear cell lines including hair cells, stria vascularis and neurons with ion channels can modulate the expression and function of K^+^ channels and co-transporters in vitro, and will help to elucidate the roles they play in aging and regulation of auditory function. Because some of these ion channel mutations are dominantly inherited in cases of deafness, acute viral introduction of dominant negative constructs may prove particularly useful in elucidating the functions of ion channels and transport proteins in hearing loss and deafness. Overall, a detailed understanding of their involvement in acquired hearing loss, such as presbycusis, would open up exciting new avenues in our mechanistic understanding of hearing impairment and could lead to new biomedical and technological therapies in this area.
